# Impact of the COVID-19 pandemic on cancer diagnoses, oncological care and cancer patients in Germany: a report from the “COVID & Cancer” workshop 2023 of the German Society for Epidemiology (DGEpi)

**DOI:** 10.1007/s00432-024-06019-3

**Published:** 2024-11-09

**Authors:** Friederike Erdmann, Maike Wellbrock, Karina Karolina De Santis, Joachim Hübner, Sven Voigtländer, Volker Arndt

**Affiliations:** 1 Research Group Aetiology and Inequalities in Childhood Cancer, Division of Childhood Cancer Epidemiology, Institute of Medical Biostatistics, Epidemiology and Informatics (IMBEI), University Medical Center of the Johannes Gutenberg University, Langenbeckstraße 1, 55131 Mainz, Germany; 2 Department of Prevention and Evaluation, Leibniz Institute for Prevention Research and Epidemiology – BIPS, Bremen, Germany; 3 Agency for Clinical Cancer Data of Lower Saxony, Oldenburg, Germany; 4 Bavarian Cancer Registry, Bavarian Health and Food Safety Authority, Nuremberg, Germany; 5 Epidemiological Cancer Registry Baden-Württemberg & Research Group Cancer Survivorship, German Cancer Research Center, Heidelberg, Germany

**Keywords:** COVID-19 pandemic, Germany, Adverse consequences, Delayed diagnosis, Incidence, Cancer care

## Abstract

**Purpose:**

The COVID-19 pandemic was associated with severe disruptions in healthcare worldwide. Cancer patients are at particular risk of adverse consequences from delays in diagnosis and treatment. To evaluate the available data on the impact of the pandemic on cancer diagnoses, oncological care and patient well-being in Germany, the German Society for Epidemiology (DGEpi) in collaboration with the Epidemiological Cancer Registry of Lower Saxony invited to a workshop on “COVID & Cancer” (held on 26–27 October 2023 in Hanover, Germany). This report provides a summary of the scientific presentations, highlights methodological challenges, and recognises essential evidence gaps.

**Methods:**

Twelve studies addressing various aspects in relation to cancer diagnoses, oncological care and patient well-being during the COVID-19 pandemic in Germany and two talks sharing experiences from the UK and the Netherlands were presented at the workshop.

**Results and conclusions:**

Results from German cancer registries consistently showed lower number of incident cancer diagnoses among adults during the first months of the pandemic compared to the respective months of the years before the pandemic. Data from the cancer registries of Baden-Württemberg and Lower Saxony found especially for breast cancer a notable drop (by approximately one third) in the numbers of diagnoses during the first restriction period (April-May 2020), during which the nationwide mammography screening programme in Germany was temporarily suspended. Overall, the extent and ways, in which the pandemic had adversely affected cancer diagnoses, oncological care and created service backlogs, is still not adequately understood. The long-term consequences are yet to be determined.

## Introduction

Following the outbreak of the novel severe acute respiratory syndrome coronavirus 2 (SARS-CoV-2 which causes coronavirus disease 2019 (COVID-19)) pandemic in early 2020, wide-ranging societal restrictions, stay-at home policies, and other public health measures were imposed across Germany and worldwide. Particularly in the beginning of the pandemic and during the subsequent COVID-19 waves, when intensive care units were severely overwhelmed with COVID-19 patients, healthcare authorities in many countries advised to postpone care for non-acute or not life-threatening conditions and temporarily suspended cancer screening programmes (Dinmohamed et al. 2020a; Teglia et al. 2022). As a result, healthcare for non-communicable diseases faced significant disruptions, with cancer patients being particularly vulnerable to the adverse consequences of delays in diagnosis and treatment. Today, an increasing number of international publications report a remarkable decline in new cancer diagnoses, evidence of missed and delayed diagnoses, and disruptions in cancer treatment since the onset of the pandemic, raising serious concerns of rising cancer mortality (Kluge 2022; Skovlund et al. 2021; The Lancet 2021; Maringe et al. 2020; Kirby 2024; Wells and Galvani 2022; Malagón et al. 2022).

Although the COVID-19 pandemic is fortunately no longer dominating the daily news, its unprecedented adverse impact on timely diagnosis and cancer care remains a significant concern (Kirby 2024). Given the large heterogeneity in welfare systems and organisation of healthcare as well as the considerable differences in the course of the pandemic and related public health measures, not all countries have experienced similar levels of disruptions. Thus, sizable differences in the adverse consequences of the COVID-19 pandemic on cancer diagnoses and care are anticipated across countries worldwide (Kirby 2024), making comprehensive, country-specific assessments of the pandemic’s impact essential.

To evaluate the available information on the impact of the pandemic on cancer diagnoses, oncological care and patient well-being in Germany, the Cancer Epidemiology Working Group of the German Society for Epidemiology (Deutsche Gesellschaft für Epidemiologie e.V., DGEpi) in collaboration with the Epidemiological Cancer Registry of Lower Saxony invited to a workshop on “COVID & Cancer”. The workshop was held on 26–27 October 2023 in Hanover, Germany and was attended by 35 epidemiologists and public health scientists from all over Germany. In addition, two speakers from the UK and the Netherlands were invited to share the experiences from their respective countries.

With this report, we provide a summary of the scientific presentations, highlight methodological challenges, and point out some essential evidence gaps that need to be addressed to reduce barriers to health care in future public health crises.

## Summary of the scientific presentations

### Scientific evidence from Germany

Overall, twelve studies addressing various aspects in relation to cancer diagnoses, oncological care and patient well-being during the COVID-19 pandemic in Germany were presented at the workshop in Hanover (Table 1).

**Table 1:** Overview of the scientific presentations at the “COVID & Cancer” workshop of the German Society for Epidemiology (Deutsche Gesellschaft für Epidemiologie, DGEpi) in collaboration with the Epidemiological Cancer Registry of Lower Saxony, 26–27 October 2023 in Hanover, Germany

Speaker	Topic/ Outcome	Study design	Data source	Study population	Study period	Cancer diagnoses
**Scientific evidence from Germany**						
De Santis, K.	Any (general or unspecified) healthcare, diagnosis, treatment, aftercare, other (specific) care	Rapid review	77 publications (59 peer-reviewed studies and 18 reports)	General population (any age), including specifically individuals eligible for screening programs in Germany, healthcare professionals in oncology	2020–2022 vs. <2020	Any
Doege, D.	Anxiety and depression in the context of contact and oncological care restrictions during the COVID-19 pandemic	Cross-sectional survey	Patient-reported outcomes via self-report questionnaires, Epidemiological Cancer Registry Baden-Württemberg (information on cancer cases)	Cancer patients and survivors (diagnosed 7/2015-6/2020), aged 18–85 years, place of residence in Baden-Württemberg, identified from the Epidemiological Cancer Registry Baden-Württemberg	2020–2021	Breast, colorectal, prostate, lung cancer or leukaemia/ lymphoma
Erdmann, F.	Absolute numbers of newly diagnosed cancer diagnoses, age-standardised incidence rates	Time-series analysis	German Childhood Cancer Registry (cancer cases), Federal Statistical Office (population estimates)	Children (Germany), aged 0–17 and 0–14 years	2020–2021 vs. 2015–2019	Any
Frick, J.	Frequency of changes in cancer care and therapy, health-related quality of life	Cross-sectional survey	Patient-reported outcomes via self-report questionnaires, Epidemiological Cancer Registry Baden-Württemberg (information on cancer cases)	Cancer patients and survivors (diagnosed 7/2015-6/2020), aged 18–85 years, place of residence in Baden-Württemberg, identified from the Epidemiological Cancer Registry Baden-Württemberg	2020–2021	Breast, colorectal, prostate, lung cancer or leukaemia/ lymphoma
Groeneveld, A.	Absolute numbers of newly diagnosed cancers, number of operations, days between diagnosis and first tumour-relevant operation	Time-series analysis	Clinical Cancer Registry Lower Saxony	Cancer patients, aged ≥ 18 years, diagnosed and/or treated in Lower Saxony	01/2019-12/2021	Any (excluding non-melanotic skin cancer), breast
Hübner, J.	Excess mortality	Time-series analysis	Epidemiological Cancer Registry Lower Saxony (cancer cases), Federal Statistical Office (population estimates)	General population of Lower Saxony with a history of cancer (no age restriction)	04/2020-03/2021 vs. 04/2016-03/2020	Any
Jansen, L.	Age-standardised incidence rates, time to surgery, type of surgery	Time-series analysis	Epidemiological Cancer Registry Baden-Württemberg (breast cases), Federal Statistical Office (population estimates)	Female population of Baden-Württemberg	2020–2021 vs. 2018–2019	Invasive and in-situ breast cancer
Kraywinkel, K.	Absolute numbers of newly diagnosed cancer diagnoses, cancer incidence, tumour stage distribution, survival, mortality, number of cancer-related surgical procedures	Time-series analysis	German Centre for Cancer Registry Data, Institute for the Hospital Remuneration System (InEK Institute), Statutory Health Insurance data (BfArM)	General population of Germany, aged ≥ 18 years	2020–2022 vs. 2016–2019 (varying by data source)	Any (excluding non-melanotic skin cancer)
Pritzkuleit, R.	Absolute numbers of newly diagnosed cancers, age-standardised incidence rates by cancer type and stage	Time-series analysis	Cancer Registry Schleswig-Holstein (cancer cases), Federal Statistical Office (population estimates)	General population of Schleswig-Holstein	2017/2018–2022	Any (excluding non-melanotic skin cancer)
Schnoor, M.	Absolute number of treatment changes	Cross-sectional study	Patients from UKSH, general practitioners, participants of the COVIDOM-Study	Adults with a cancer diagnosis in the past 5 years and a confirmed SARS-CoV-2 infection	03/2020-05/2022	Any (excluding non-melanotic skin cancer)
Schweiger, L.	Absolute number of newly diagnosed cancer diagnoses, stage distribution, crude incidence rates, age-standardised incidence rates	Time-series analysis	Epidemiological Cancer Registry of Lower Saxony (cancer cases), State Office for Statistics, Lower Saxony (population estimates)	Adult population of Lower Saxony, aged ≥ 18 years	2020–2021 vs. 2015–2019	Any (excluding non-melanotic skin cancer); 10 selected common cancer sites
Wellbrock, M.	Overall survival, event-free survival	Time-series analysis	German Childhood Cancer Registry (cancer cases), population registries (vital status)	Children (Germany), aged 0–14 years	2020–2022 vs. <2020	Any
**Experiences from the UK and the Netherlands**						
McPhail, S. (UK)	Rapid registration and monitoring of new cancer diagnoses, treatment		National Cancer Registration Data; Rapid Cancer Registration	General population of England	1st quarter 2018–1st quarter 2023	Any
Siesling, S. (The Netherlands)	(Relative) changes in absolute numbers of newly diagnosed cancer diagnoses, stage distribution, incidence rates, screening, primary treatment of new cancer diagnoses and end of life care	Time-series analysis	The Netherlands Cancer Registry	General population of the Netherlands	Calendar weeks of 2020; 2020 vs. 2018 & 2019	Any

*Abbreviations* DGEpi, Deutsche Gesellschaft für Epidemiologie (German Society for Epidemiology); UKSH, Universitätsklinikum Schleswig-Holstein (University Hospital Schleswig-Holstein); InEK Institute, Institut für das Entgeltsystem im Krankenhaus (Institute for the Hospital Remuneration System); BfArM, Bundesinstitut für Arzneimittel und Medizinprodukte (Federal Institute for Drugs and Medical Services)

#### Changes in the number of diagnoses and cancer incidence rates

The registration of cancer diagnoses in adults in Germany is organised at the federal state level. Results from three different statewide, population-based cancer registries, as well as from the nationwide German Childhood Cancer Registry, evaluating changes in newly diagnosed cancer cases and incidence rates during the pandemic in Germany, were presented at the workshop. The state cancer registries consistently reported either lower incidence rates or a reduced number of diagnoses for all cancer types combined among adults during periods of heightened pandemic restrictions (particularly in April-May 2020) compared to the respective months in pre-pandemic years (Pritzkuleit and Katalinic 2023; Groeneveld et al. 2023; Schweiger et al. 2023; Jansen 2023; Jansen et al. 2024). Whereas in Lower Saxony the age-standardised incidence rate (ASR) for all cancer sites combined in 2020 was 5% lower relative to the pre-pandemic years (Schweiger et al. 2023), the cancer registry of Schleswig-Holstein did not report any pronounced decline in the annual incidence rates, but nonetheless a marked drop in the number of new cancer diagnoses in April 2020 (Pritzkuleit and Katalinic 2023). It was assumed that the drop in new cancer diagnoses in April 2020 (i.e. during the first high restriction period in Germany) was caught up once the restrictions were eased based on higher numbers of monthly diagnoses after May 2020. Data from the cancer registries of Baden-Württemberg and Lower Saxony showed a notable drop in cancer incidence for a longer period. Particularly for breast cancer, a drop by approximately one third during the first restriction period (April-May 2020) was reported, which may likely be a consequence of the nationwide temporary (five-week) suspension of the breast cancer screening programme (Jansen 2023; Schweiger et al. 2023; Jansen et al. 2024). An exception were the higher incidence rates observed in Lower Saxony in 2020 for pancreas carcinomas (+ 5.9%) and cervical cancer (+ 4.6%) (Schweiger et al. 2023).

A further presentation summarised the results of a rapid review, which systematically and comprehensively assessed the impact of the COVID-19 pandemic on oncological care in Germany (De Santis et al. 2023). The review showed a drop in new cancer diagnoses especially during the first restriction period based on data from several cancer registries that were not individually addressed at the workshop (De Santis 2023; De Santis et al. 2023).

Contrary to the widely reported decrease in adult cancer diagnoses in 2020, a marked increase in the incidence of childhood cancer was observed in 2020 compared to the five years before the pandemic (2015–2019). Analysis of data from the nationwide German Childhood Cancer Registry revealed an increase in incidence rates of 8–12% across all diagnostic groups (defined according to the International Classification of Childhood Cancer) (Steliarova-Foucher et al. 2005), followed by a decrease for most cancer types in 2021 compared to 2020 (Erdmann 2023; Erdmann et al. 2021). However, estimated ASRs of childhood cancer overall, leukaemia overall, lymphoid leukaemia, and non-malignant central nervous system (CNS) tumours in 2021 were still somewhat elevated compared to those in the pre-pandemic period. In contrast, ASRs for lymphomas and non-CNS solid tumours have dropped so drastically that they fell below the respective ASRs in 2015–2019 (Erdmann 2023; Erdmann et al. 2021). Although it is reassuring that there is little evidence for missed or delayed childhood cancer diagnoses throughout 2020–2021, the underlying reasons for the marked increase in incidence rates in 2020 remain speculative. An actual increase in risk for childhood cancer overall in direct or indirect response to the COVID-19 pandemic appears highly implausible according to the current scientific knowledge, but seems conceivable for B-cell precursor acute lymphoblastic leukaemia, the most common subtype of paediatric acute lymphoblastic leukaemia (ALL). For childhood ALL specifically, an aetiological link to patterns of exposure to infections and immune system stimulation has been hypothesised (Hauer et al. 2021; Greaves 2018; Schüz et al. 2022). The underlying reasons—at least for the patterns observed in the incidence of solid tumours—may include increased parental attention and greater physician awareness of early disease symptoms, leading to more timely healthcare consultations and earlier diagnoses.

#### Changes in cancer stage

The cancer registry of Schleswig-Holstein reported no shift in tumour stages during 2020 (Pritzkuleit and Katalinic 2023). In contrast, a marked difference in the distribution of tumour stages in 2020 (relative to 2015–2020) was evident among people with lung cancer in Lower Saxony, where diagnoses of T1 and T4 stages increased and T2 and T3 stages decreased (Schweiger et al. 2023). However, since no further data from other regions in Germany were reported, it remains unknown at this stage to which extent changes in cancer stage have occurred nationwide in Germany.

#### Changes in oncological care

Overall, 25% and 75% of cancer patients who participated in survey studies in Baden-Württemberg and Schleswig-Holstein, respectively, reported perceived changes in their initial treatment in 2020–2021 (Frick 2023; Schnoor et al. 2023). In Baden-Württemberg, the most common changes were related to rehabilitation, follow-up care, and surgery (no information was available for Schleswig-Holstein at the time of the workshop, as analyses were still ongoing). According to the rapid review by De Santis et al., other treatments than surgery (e.g., psychosocial care and aftercare) were less often addressed in published studies in Germany and any disruptions in these treatments were reported less consistently. Any ongoing therapies and cancer care for advanced or high-risk cases remained mostly unaffected (De Santis 2023; De Santis et al. 2023). For breast cancer, the strongest drop in the number of surgeries occurred in May (by approximately a third compared to the previous month, April 2020) and June 2020 (by approximately 20% compared to April 2020) in Lower Saxony (Groeneveld et al. 2023). However, time to surgery in breast cancer patients remained relatively constant during the pandemic relative to pre-pandemic years in Baden-Württemberg, while a slight increase in the proportion of mastectomies was observed in 2021 (Jansen 2023; Jansen et al. 2024). In Lower Saxony, surgeries for all tumour types – and breast cancer in particular – diagnosed in March and April 2020 were performed within a shorter time compared to other months in 2020 and the pre-pandemic periods (Groeneveld et al. 2023).

#### Changes in cancer survival and mortality in cancer patients

Cancer survival and mortality were addressed in three presentations at the workshop (Hübner et al. 2023; Kraywinkel 2023; Wellbrock 2023). Using data from Lower Saxony, one study compared the pandemic-associated excess mortality in individuals with a history of cancer to the excess mortality in the general population (Hübner et al. 2023). Relative to the pre-pandemic period, the excess mortality was 6.6% in the cancer survivors compared to 3.3% in the general population. This pattern was most pronounced among the elderly (aged 75+ years) with an excess mortality of 9.5% vs. 3.8% and in females (5.4% vs. 1.4%). Since no further mortality data were reported from other regions in Germany, it is unclear whether these results are generalisable nationwide. Further presentations described two planned research projects that aim to assess cancer survival in children (Wellbrock 2023) and cancer survival and mortality (in addition to other outcomes, including incidence, tumour stage distribution and treatment on a nationwide scale) in adults aged ≥ 18 years (Kraywinkel 2023) during the COVID-19 pandemic relative to any pre-pandemic period (Wellbrock 2023) or 2016–2019 (Kraywinkel 2023).

#### Changes in mental health and health-related quality of life in cancer patients and survivors

Mental health and quality of life associated with oncological care and treatment changes during the pandemic were reported in two presentations based on a survey of adult cancer patients or survivors in Baden-Württemberg (Doege 2023; Frick 2023; Doege et al. 2024). Changes in care and treatment were associated with impaired mental well-being (i.e., increased severity of anxiety and depression symptoms) (Doege 2023; Doege et al. 2024) and reduced global quality of life in multiple domains, including physical, cognitive, and psychosocial functioning (Frick 2023). Besides individual factors, such as age < 60 years, female sex and low income, contact restrictions to peer support groups, relatives, physicians and caregivers were identified as risk factors for increased severity of anxiety and depression symptoms (Doege 2023; Doege et al. 2024). The rapid review by De Santis et al. (De Santis 2023; De Santis et al. 2023) also highlighted that most studies on cancer care during the COVID-19 pandemic in Germany focused on incidence and timeliness of diagnoses and treatment, while only a minority of studies addressed mental well-being of patients and survivors or survival rates.

### Experiences from the UK and the Netherlands

To enrich the workshop, two international speakers were invited. Sean McPhail (National Disease Registration Service, NHS England, UK) and Sabine Siesling (Netherlands Comprehensive Cancer Organisation (IKNL)) shared their experiences and perspectives from the UK and the Netherlands, respectively (McPhail et al. 2023; Siesling 2023).

In the UK, in response to the need for a near real-time monitoring of cancer incidence and care during the COVID-19 pandemic, a system called “Rapid Cancer Registration Data” (RCRD) was introduced by NHS England and the National Disease Registration Service (NDRS) (NHS England and National Disease Registration Service 2024). The system is based on various administrative hospital-based data (i.e. hospital cancer patient management systems, hospital patient administration systems, and hospital cancer referral speed tracking systems) as well as mortality data from the national death certificate information. All these data arrive at NDRS within four months of the initial documentation, and capture about 90–95% of all cancer diagnoses when compared to the regular cancer registration data available 21 months post-diagnosis. RCRD contains proxy tumour registrations and some associated events on the cancer care course (e.g. surgery, radiotherapy and chemotherapy) from January 2018 to the most recent date of data availability (NHS England 2018). According to *Sean McPhail*, RCRD provides a reasonable proxy for cancer registrations, but misses up to 10% of all registrations (varying by age and tumour type) and includes a less complete information regarding tumour stage (McPhail et al. 2023).

The Netherlands Cancer Registry report was one of the first publications to document a drop in cancer diagnoses during the COVID-19 pandemic, with its findings published already on 4th May 2020 (Dinmohamed et al. 2020b). *Sabine Siesling* from the Netherlands Comprehensive Cancer Organisation (IKNL) provided an overview of the various COVID-19 pandemic research activities based on data from the Netherlands Cancer Registry. The research conducted by IKNL aimed to offer concrete recommendations for policy, for effective communication to the general population as well as primary and secondary care to ensure continued effective diagnosis and care for cancer patients during the pandemic (Siesling 2023). Specifically, they addressed the impact of the COVID-19 pandemic on the diagnosis and treatment of various cancer types, the effects of the temporary suspension of cancer screening programmes in the Netherlands as well as potentially unsuitable end of life care during the pandemic. Same as in Germany, cancer diagnoses declined due to reluctance of patients to seek healthcare, prolonged diagnostic pathways and the temporary suspension of the screening programmes. However, the number of cancer diagnoses had mostly caught up by November 2023. This catch-up effect was supported by a campaign to urge patients with complaints to visit their general practitioner and the gradual restart of the screening programmes. In addition, adapted treatment protocols were rapidly implemented, and video consultations were frequently used in clinical practice. In summary, all stakeholders (healthcare providers, patient organisations and policymakers) managed to respond to the pandemic quickly, subsequently limiting the impact on oncological care. However, the possible effect of the delay in diagnoses on tumour stage and prognosis needs close monitoring and further evaluation in the future (Siesling 2023).

### Methodological challenges

#### Direct versus indirect impact of the COVID-19 pandemic

When evaluating the impact of the COVID-19 pandemic on the cancer care trajectory, two overall objectives should be considered: First, what diagnosis- or treatment-related consequences arise in individuals with cancer who were affected by a COVID-19 infection? Second, which adverse consequences of the COVID-19 pandemic have been observed at the level of the general population?

The first objective was addressed in only one presentation at the workshop (Schnoor et al. 2023) that aimed to evaluate treatment changes among cancer patients with a confirmed SARS-CoV2 infection. However, only a very small number of patients were identified and included in the study, suggesting a considerable risk of selection bias. The majority of studies presented at the workshop addressed the second objective in that the consequences of the pandemic were examined on the population level using data from population-based cancer registries.

#### Timing of cause and effect

Hypothesis-driven analyses of causal relationships via time series analyses require accurate assumptions about the timing of both the potential cause and the potentially associated event. Defining the period of high restrictions is straightforward for some scenarios, such as a nationwide suspension of a screening programme that took place for the mammography screening for a well-documented five-week period in March to April 2020 in Germany. In contrast, other scenarios do not have a well-documented timing and thus their causation is difficult to determine. One example concerns the decreased healthcare seeking behaviour (including screening) during highly variable levels of restrictions in public life (e.g. contact restrictions) that were temporarily introduced in different regions at different times in Germany.

Another uncertainty relates to the expected lag time between missed healthcare services and their impact on the outcome of interest (Gierz et al. 2022). The suspension of the national mammography screening programme has certainly caused a substantial drop in the number of diagnoses within a short-term of only few weeks, whereas any increases in diagnoses with advanced stage or mortality can only be observed in a long-term perspective (Lee et al. 2013). Furthermore, the duration of the expected effect is hard to anticipate. Any underuse of adjuvant therapy lasting a few months, for example, is expected to cause recurrences over a period of subsequent several years.

The uncertainty in definition of high restriction periods, lag time of relevant outcomes, and duration of effects make it difficult to define appropriate comparison periods for statistical testing. Using annual data may miss effects of short duration, whereas focusing on monthly data can be affected by statistical noise caused by a small number of events. However, most of the studies presented at the workshop did not include formal inferential statistical testing. One study analysed monthly incidence data for several cancer sites using Joinpoint regression analysis (Pritzkuleit and Katalinic 2023). Visually noticeable drops in the number of diagnoses in April and May 2020, however, remained undetected by the regression model. A subsequent debate among the workshop participants questioned whether Joinpoint regression analysis, which was developed to detect points in time marking significant changes in magnitude or direction of temporal trends (the so-called joinpoints) (Kim et al. 2000), is a suitable statistical approach to detect such short-term deflections of a trend, particularly given the small sample sizes.

#### Competing risks and estimation of expected events

Attributing research findings causally to the pandemic typically involves direct comparisons with pre-pandemic patterns. The presentation by Hübner et al. emphasised that the outcomes of their study on excess mortality were highly dependent on how expected events were estimated—either as averages of pre-pandemic years or modelled through linear regression. Both methodological approaches may be applied to absolute numbers of cases, crude rates, or age-standardised rates. The choice of method influences the extent to which temporal trends, such as demographic changes or changes in risk, are accounted for. In addition, the length and definition of the pre-pandemic reference period need to be considered. In the studies presented at the workshop, the start of the pre-pandemic reference periods varied from 2015 to 2018.

Regarding both the drop in incidence rates and the incidence of advanced-stage tumours, it is possible that some of the “missed diagnoses” would have never occurred because the potentially affected individuals died from competing causes (including COVID-19 infections) before the cancer could develop or be detected. This is a plausible scenario, as the COVID-19 pandemic primarily caused deaths among individuals who were also at elevated cancer risk, such as the elderly, smokers, obese individuals, or those with diabetes. The result is a selective reduction in the population with an elevated cancer risk, leading to a true reduction in the overall cancer burden in the general population. This selective reduction must be distinguished from a decline in estimated incidence rates due to underdiagnoses.

#### Timeliness of data availability

Meaningful time-series analyses require high data quality and completeness over the entire study period to detect any reductions and catch-up effects over time. Normally, the completion of ascertainment of incidence records takes up to approximately two years in Germany, with some variation between individual cancer registries. Therefore, the complete data for the COVID-19 pandemic period was not available in Germany for all pandemic years at the time of the workshop (October 2023). It is also unclear to what extent the changes in healthcare workload during the pandemic may have influenced fulfilment of reporting obligations and the quality of available data.

### International context

A vastly growing number of international assessments on the impact of the COVID-19 pandemic on cancer diagnoses, cancer screening tests, diagnostic procedures and provision of oncological care, has been published after the outbreak of the COVID-19 pandemic in March 2020. One of the first such publications included the report from the Netherlands Cancer Registry that noted a considerable drop in new cancer diagnoses during the first months of the COVID-19 pandemic and a modelling study on the consequences of delays in surgery for cancer in the UK (Dinmohamed et al. 2020b; Sud et al. 2020). Subsequently, numerous international studies assessed a wide range of different outcomes, in particular screening rates, pathology notifications, number of new cancer diagnoses, changes in the distribution of tumour stages and also disruptions in treatment and the incidence of COVID-19 infections in cancer patients (Bakouny et al. 2020; Voigtländer et al. 2023; Angelini et al. 2023). Overall, large reductions in diagnostic tests for cancer and the number of new cancer diagnoses for all major cancer sites during the first COVID-19 wave were reported from various European countries and the USA, often followed by indications of rebound effects that did not reach the pre-pandemic levels (Ribes et al. 2022; Johansson et al. 2022; Peacock et al. 2021; Morris et al. 2021; Kaufman et al. 2021; Angelini et al. 2023). Moreover, several studies found, in addition to a drop in the number of newly diagnosed cancers, a greater proportion of late stage cancer diagnoses during 2020 (Angelini et al. 2023).

From an international perspective, comparing cancer incidence across European countries and North America indicated considerable differences in the relative reduction of ASRs for cancer in 2020 compared to 2019, ranging from 3% in Austria to 13% in the UK with most pronounced reductions for prostate cancer, whereas reductions for other tumour sites were more heterogeneous (Kraywinkel et al. 2024). Generally, the impact of the COVID-19 pandemic in Germany appears to have been less pronounced than in the USA, that in turn experienced a lower impact of the pandemic on cancer than the UK (Kirby 2024). For outcomes other than the number of cancer diagnoses, such as shifts in stage and treatment disruptions, comprehensive assessments from Germany are still lacking, which hinders international comparisons.

For childhood cancers specifically, the results from the data of the German Childhood Registry were not in line with most international observations. The few reports addressing the impact of the COVID-19 pandemic on childhood cancer incidence indicated mostly fewer newly diagnosed paediatric cancers than expected, although observations were not fully consistent. Observations ranged from an overall stable number of children diagnosed with cancer (Kourti et al. 2020), to substantially fewer diagnoses than expected (O’Neill et al. 2020; Chiaravalli et al. 2020), especially in some diagnostic groups or cancer types but not in others (Offenbacher et al. 2020; Jarvis et al. 2021; Ding et al. 2020). However, the available evidence presents only a fragmented picture, limited by respectively small sample sizes, frequently of single institutional or regional nature, and capturing only the period of the first pandemic wave in 2020.

International differences regarding the impact of the COVID-19 pandemic on cancer diagnoses and care are in general not yet well described. However, given the large heterogeneity in welfare and healthcare systems, including organisation of healthcare, social and cultural differences, as well as the sizable differences in the pandemic course and resulting public restrictions, pronounced differences in the adverse consequences of the COVID-19 pandemic across countries are strongly anticipated. For example, the temporary suspension and subsequent reorganisation of screening programmes, as well as public campaigns urging patients to attend screening and seek medical care in case of complaints (Dinmohamed et al. 2020a; Voigtländer et al. 2023) differed markedly across countries. Furthermore, pandemic preparedness, which was associated with lower excess mortality (Ledesma et al. 2023) and presumably a lower impact of the COVID-19 pandemic on cancer care, also differed across countries.

### Evidence gaps

The pandemic has affected the entire cancer care trajectory, not only due to changes in health system capacity but also due to the substantial changes in healthcare seeking behaviour. The extent and ways in which the pandemic delayed cancer care and created service backlogs is, however, not adequately understood and important questions remain unanswered, not only in Germany but across countries worldwide. Also the recently published findings from three new population-based assessments (each based on data from different statewide cancer registries), published after the workshop, do not alter the overall picture for Germany (Carré et al. 2024; Brenner et al. 2024; Reinwald and Justenhoven 2023). In general, the evidence gaps range from nationwide, population-based systematic assessments of delayed and missed cancer diagnoses, shifts in tumour stages, and treatment disruptions to psychosocial care and aftercare, including regional differences within Germany. There is particularly limited evidence on the mental burden, quality of life, and well-being of patients and cancer survivors, as well as on the most affected individuals and vulnerable groups, and the specific underlying mechanisms driving these unfavourable consequences of the pandemic. Furthermore, the consequences beyond 2020 are especially poorly understood. The wide-ranging adverse implications of the COVID-19 pandemic are certainly best understood for the months of the first wave in 2020, but evidence regarding disruptions during the subsequent waves is sparse and there is yet not enough data to assess adverse consequences in relation to the delta and omicron waves in 2021 and 2022 (Kirby 2024). A recent publication based on data from the Saarland Cancer Registry found indications of a sustained delay in colorectal cancer diagnoses in Germany until at least mid-2021, and likely beyond (Brenner et al. 2024). However, these observations relate solely to one cancer type and are based on data from a single, small region of Germany. The patterns for other cancer types and regions across the country remain unclear at this point.

Irrespective of data availability, a comprehensive assessment regarding the full adverse implications of the pandemic is possible only after sufficient time has passed. The long-term impact of pandemic-related cancer diagnostic and treatment delays and disruptions on mortality as well as somatic and mental health conditions will only become evident over the years and increased cancer mortality would be expected to span over several years. A meta-analysis estimated that each 4-week delay in cancer surgery could lead to a 6–8% increase in mortality across a number of major cancer sites (Hanna et al. 2020). Estimates for systemic treatment and radiotherapy were somewhat inconsistent but implied also important increases in mortality for several cancer sites. A modelling study from Canada (Malagón et al. 2022) predicted that cancer sites with moderate to high net survival and cancers in younger individuals were likely to experience the strongest relative mortality increase. Moreover, most severe treatment delays were anticipated for surgeries, which would imply a greater impact on such diagnoses that require surgery. Understanding the future public health implications would certainly help to prioritise allocation of resources to regain the pre-pandemic level of oncological care, outcome and quality of life of patients and cancer survivors.

## Conclusions and perspectives

The extent and ways in which the COVID-19 pandemic has adversely affected the entire cancer care trajectory and created service backlogs is not adequately understood and important questions remain unanswered. Findings from the 12 presentations at the “COVID & Cancer” workshop, using data from Germany indicated lower number of incident cancer diagnoses among adults during the first months of the pandemic compared to the corresponding months in previous years. The stay home-orders, the general fear of a COVID-19 infection and the advice to postpone care for non-acute conditions, along with the nationwide suspension of the mammography screening programme in April 2020 in Germany, likely contributed significantly to the decline in cancer diagnoses. Data from the cancer registries of Baden-Württemberg and Lower Saxony found a notable drop in the numbers of new breast cancer diagnoses during the first restriction period (April-May 2020), during which the nationwide mammography screening programme was suspended in Germany.

Results from the workshop suggest that regional differences within Germany not only pertained to the course of the pandemic and corresponding public health restrictions, but also to the unfavourable consequences of the COVID-19 pandemic, which varied markedly across federal states and likely at more localised regional levels as well. However, nationwide, population-based, systematic assessments including evidence on regional differences within Germany are still lacking. In international comparison between high-income countries, the evidence available in Germany seems to be sparse. This is likely a result of delays in data availability due to the regional rather than central organisation of cancer registration for adult cancer in Germany that is done on the level of the 16 federal states in Germany and the interval before cancer registry data of sufficient quality is available. The newly implemented electronic health record on the insurance card and the Health Data Utilisation Act (GDNG) will offer new possibilities for a timely and more comprehensive monitoring of outcomes in cancer patients. Alternatively, supplemental use of hospital discharge records might allow more timely monitoring as demonstrated by the Rapid Cancer Registration Data in England.

Irrespective of data availability, a comprehensive assessment regarding the full implications of the pandemic is, however, only possible after sufficient time has passed and at this point no final conclusions can be drawn. The long-term impact of pandemic-related cancer diagnostic and treatment delays and disruptions on mortality as well as somatic and mental health conditions will only become evident over the coming years. A more profound understanding of the adverse effects of health policies during the pandemic is central to guide policymaking and mitigate barriers to healthcare in future public health crises.

## Data Availability

The slides of the individual scientific presentations at workshop can be found at: https://www.dgepi.de/de/arbeitsgruppen/AG/8.
